# Visual dysfunction in dementia with Lewy bodies

**DOI:** 10.1007/s11910-024-01349-8

**Published:** 2024-06-22

**Authors:** Ryan A. Devenyi, Ali G. Hamedani

**Affiliations:** 1 Department of Neurology, Perelman School of Medicine, University of Pennsylvania, Philadelphia, PA USA; 2 Department of Ophthalmology, Perelman School of Medicine, University of Pennsylvania, Philadelphia, PA USA; 3 Department of Biostatistics, Epidemiology, and Informatics, Perelman School of Medicine, University of Pennsylvania, Philadelphia, PA USA

**Keywords:** Dementia with Lewy bodies, Mild cognitive impairment, Visual impairment, Visuoperception, Retina, Visual cortex

## Abstract

**Purpose of Review:**

To review the literature on visual dysfunction in dementia with Lewy bodies (DLB), including its mechanisms and clinical implications.

**Recent Findings:**

Recent studies have explored novel aspects of visual dysfunction in DLB, including visual texture agnosia, mental rotation of 3-dimensional drawn objects, and reading fragmented letters. Recent studies have shown parietal and occipital hypoperfusion correlating with impaired visuoconstruction performance. While visual dysfunction in clinically manifest DLB is well recognized, recent work has focused on prodromal or mild cognitive impairment (MCI) due to Lewy body pathology with mixed results. Advances in retinal imaging have recently led to the identification of abnormalities such as parafoveal thinning in DLB.

**Summary:**

Patients with DLB experience impairment in color perception, form and object identification, space and motion perception, visuoconstruction tasks, and illusions in association with visual cortex and network dysfunction. These symptoms are associated with visual hallucinations, driving impairment, falls, and other negative outcomes.

## Introduction

Dementia with Lewy bodies (DLB) is the second-most common neurodegenerative cause of dementia after Alzheimer disease (AD) and is defined pathologically by the presence of synuclein-positive Lewy bodies [1–3]. In addition to progressive cognitive decline leading to dementia, DLB is characterized by four cardinal symptoms: fluctuating cognition, recurrent visual hallucinations, rapid eye movement (REM) sleep behavior disorder (RBD), and parkinsonism [4] (Table 1). Cognitive impairment in DLB is most prominent in the executive, attention and visuospatial domains, with relative sparing of memory earlier in the disease [4–6]. Prominent visuoperceptive dysfunction and visual hallucinations figure prominently in the most recent diagnostic criteria for DLB (Table 1) [4], In this article, we review the patterns, causes, and clinical implications of visual dysfunction in DLB.

**Table 1:** Summary of 2017 Revised criteria for the clinical diagnosis of dementia with Lewy bodies [4]

**Essential Feature** •Dementia, potentially with prominent impairment in attention, executive function, and visuoperception	
**Core Clinical Features** • Fluctuating cognition • Recurrent visual hallucinations, typically well-formed • Rapid eye movement (REM) sleep behavior disorder • Parkinsonism	
**Supportive Clinical Features** • Antipsychotic sensitivity • Postural instability • Repeated falls • Syncope or other transient episodes of unresponsiveness • Severe autonomic dysfunction • Hypersomnia • Hyposmia • Hallucinations in non-visual modalities • Systematized delusions • Apathy, anxiety, and depression	
**Indicative biomarkers** • Reduced basal ganglia dopamine transporter uptake (SPECT or PET imaging) • Reduced cardiac MIBG scintigraphy • Confirmation of REM sleep without atonia (polysomnography)	
**Supportive biomarkers** • Relative preservation of mesial temporal structures (CT or MRI) • Reduced occipital activity and/or cingulate island sign (FDG-PET) • Prominent posterior slow-wave activity with periodic fluctuations in pre-alpha/theta range (EEG)	
**Probable DLB** can be diagnosed with either: • >2 clinical features • 1 core clinical feature + >1 indicative biomarkers	
**Possible DLB** can be diagnosed with either: • 1 core clinical feature • 0 core clinical features + >1 indicative biomarkers	

### Overview of the central visual pathways

The retina converts light into signals that are relayed by white matter tracts to the lateral geniculate nucleus and ultimately the visual cortex. Cortical visual processing begins in the primary visual cortex (V1) and secondary visual cortex (V2) before extending to large areas of the occipital, parietal, and temporal lobes. Higher order visual processing can be conceptually divided into two broad domains: the ventral “what” and dorsal “where” streams. The ventral “what” stream involves the inferior occipital and temporal lobes and is important for generating perceptual representations from visual input, including processing color, form, shape, and recognizing faces and objects. The dorsal “where” stream is located in the superior occipital and parietal lobes and is important for perceiving spatial localization and motion, including motor planning and execution in response to visual inputs [7].

While visual acuity is typically preserved in DLB [8], the functions of the dorsal and ventral streams are both prominently affected, resulting in broad impairments in visuoperception and visuospatial function (Table 2). Consistent with these symptoms, studies have demonstrated hypometabolism, hypoperfusion, abnormal connectivity, reduced gray matter thickness, and white matter disruption within the occipital lobes [4, 9–19]. However, pathological studies have found that the occipital lobe is not disproportionately affected by Lewy body density or neuronal loss compared to other cortical regions in DLB [20–24]. The occipital cortex has reduced PET markers of cortical synaptic density in DLB and PDD, but this is not of sufficient amplitude to account for the degree of occipital hypometabolism seen [25]. Rather, subcortical structures with projections to the visual cortex such as the pulvinar nucleus and superior colliculus have significant pathological involvement, which may contribute to functional impairment in visual pathways and regions [26–28].

**Table 2:** Overview of vision-related cognitive deficits in DLB

- Color discrimination and contrast sensitivity	
- Object and form recognition	
- Visuospatial impairment	
- Visual agnosia	
- Pareidolia (scene and noise)	
- Visuoconstructional impairment (including figure copying)	
- Visual search impairment	

### Visual networks in DLB

A number of studies have examined alterations in network connectivity in DLB. As recently systematically reviewed by Habich *et al*., visual networks, along with frontoparietal networks and the default mode network, are together the most prominently and consistently altered networks across studies and modalities (structural, fMRI, PET, and EEG) [10], concordant with the hallmark clinical deficits in visuoperception, attention, and executive function seen in DLB. Alterations in white matter tracts connecting different regions are associated with decreased activity in associated cortical regions, and may contribute to this impaired network connectivity [29].

These network changes correlate directly with performance on neuropsychological tests of visuospatial function [9, 18, 30, 31]. For example, one study found that performance on a visual search attentive matrix task correlated with primary visual network connectivity as measured using FDG-PET connectivity, and the Raven’s colored progressive matrix task (a test of visuospatial reasoning) correlated with executive prefrontal cortex network connectivity [32]. Network alterations figure prominently in a number of visual symptoms in DLB, especially visual hallucinations, as discussed below.

### Contrast and Color

Aspects of visual function that involve perceiving shades of contrast or color may be comparatively simple compared to other higher order tasks, but the anatomy of these functions is nevertheless complex. Contrast sensitivity [33] and color vision [33–35] have both been shown to be impaired in DLB compared to controls and AD. Color vision is especially promising as a potential biomarker of DLB given its impairment in prodromal DLB and in patients with isolated RBD who later develop parkinsonism-dementia syndromes [35, 36]. To our knowledge, the prevalence of visual field defects in DLB has not been reported. Homonymous visual field defects can occur in posterior cortical atrophy, but this syndrome is more often caused by AD than DLB pathology.

Contrast sensitivity and color vision are prominently affected in retinal and optic nerve disease. As such, while research has historically focused on the brain as the primary source of visual dysfunction in DLB, there is growing evidence that the retina may also be affected. Pale retinal inclusions have been identified at autopsy in a single patient with DLB [37], and electroretinogram abnormalities have also been reported [38]. Retinal phosphorylated α-synuclein was previously reported in a subset of patients with PD (7/9) and DLB (1/3) [39], and a more recent study found retinal and optic nerve head α-synuclein aggregates and inclusions in 5/5 patients with DLB and 16/21 patients with PD [40].

Optical coherence tomography (OCT) analysis of patients with DLB has demonstrated thinning of the ganglion cell layer, but this is associated with both age and occipital lobe volume in the general population, so it was initially unclear if this represents primary retinal pathology or is a marker of age and global neurodegeneration [41–43]. However, a recent study did show significant OCT differences between age-matched groups of patients with DLB and healthy controls, including thinner ganglion cell layer and altered microvasculature [44]. In general, the retina has been better studied in PD than DLB [45–50]. More work utilizing OCT and related measures on larger DLB cohorts would be helpful for expanding on these early findings.

### Object and Form Recognition

Perception of an image’s size, shape, form, and orientation involves aspects of the dorsal and ventral streams, both of which are affected in DLB. As a result, a number of impairments in object recognition have been identified in DLB. Visuoperceptual impairments in DLB can be elicited through the use of images with distorted or missing components (such as fragmented letters), which require reconstructing an image from incomplete information. A number of studies have found impairment in object and form recognition tasks in DLB, including identifying overlapping figures, silhouettes, rotated figures, incomplete figures, and figures from illusory contours (Table 3) [33, 51–56]. The fragmented letters task may be particularly sensitive for differentiating DLB from AD [55, 57, 58]. Patients with DLB have also been shown to have impairments in tasks requiring assessment of shape features, such as matching polygons [51], analyzing the number of cubes in a shape [33, 55], and visual search for target features in an array of shapes [61, 62].

**Table 3:** Summary of studies of visual dysfunction in DLB

	Examples of tests	Summary of findings
Color vision	Farnsworth D-15 color arrangement [33], City University Color Vision Test [33], VPTA Color naming [33], NEVIP Color Discrimination [51]	Impaired compared to healthy controls and AD.
Contrast Sensitivity	Low contrast letter charts [33]	Impaired compared to healthy controls and AD.
Object identification and form or shape discrimination	Overlapping figures [52, 53], VPTA overlapping figures [54], VOSP object decision [33, 55], VOSP silhouette identification [55], illusory contours [54], Hooper visual organization test [56], NEVIP form discrimination [51], VOSP cube analysis [33, 55]	Impaired compared to healthy controls and AD.
Letter recognition	Complete letters [57], Fragmented/Incomplete letters [55, 57, 58]	Complete letters normal but fragmented letters impaired.
Texture identification	Real and image visual texture recognition [33]	Impaired compared to healthy controls and AD.
Size and spatial orientation	Line orientation discrimination [8, 54], contour integration [8], size discrimination [52], size and angle discrimination [53], NEVIP angle discrimination [51]	Impaired compared to healthy controls and AD.
Motion perception	Space and motion perception (dot position, motion perception, visual counting) [53], NEVIP car motion [51], DTVP position in space [33], dot motion perception [59].	Impaired compared to healthy controls and AD.
Mental rotation	Lego object rotation decision [8], block mental rotation task [60]	Impaired compared to healthy controls.
Visual search	Parallel and serial feature searches [61, 62]	Impaired compared to healthy controls and AD.

NEVIP: Newcastle Visuoperception Battery

VOSP: Visual object and space perception

VPTA: Visual Perception Tests for Agnosia

DTVP: Developmental Test of Visual Perception

A particular type of visuoperceptual impairment is known as visual agnosia, which refers to the inability to recognize an object despite being able to see all elements of the image. While commonly tested using 2-dimensional or 3-dimensional images of everyday household objects or representations of familiar faces, recent studies have examined the ability to recognize object textures (visual texture agnosia), finding DLB patients have impaired recognition of both real and computer-generated image textures, with particular difficulty in recognizing ceramic textures, independently of contrast sensitivity and color vision [33].

People with DLB show abnormal electrographic responses and connectivity in response to visual stimuli, including checkerboard patterns and faces. Findings include delayed visual evoked potentials, prolonged late visual responses and reduced theta activation in response to visual stimuli but not auditory stimuli, indicating that this disruption specifically involves vision-related networks [63–65]. Using resting state EEG, occipital visual networks have also been shown to be reduced in DLB, with decreased occipital alpha correlating with impaired visual shape discrimination [66]. Further work is needed to elucidate the causative role of these network disruptions in object recognition.

### Spatial Orientation and Motion

Impairment in perception of space and motion are also broadly impaired in DLB. Examples of visuospatial impairment in DLB include difficulty with mental rotation of complex images [8, 60], assessment of position in space [33, 53], and motion detection [51, 53, 59] This impairment varies with the task in question: for example, assessment of the orientation of individual lines has been found to be preserved in DLB, but determining the orientation of a 3-dimensional object is impaired [8]. Of note, visuospatial impairment in DLB is associated with executive dysfunction, cognitive fluctuations, and hallucinations, but is independent of memory impairment or global measures of cognition such as the Mini Mental Status Exam (MMSE) [60]. This suggests that visuospatial dysfunction is a manifestation of DLB-specific cognitive impairment rather than simply a reflection of global cognitive impairment.

Task-specific fMRI studies during visual motion tasks show that patients with DLB have less activation of the middle temporal visual area (V5/MT), which is involved in motion detection, compared to AD and healthy controls [67, 68]. In contrast, fMRI responses in other regions to other stimuli were not significantly different in patients with DLB, including to color, face, and pattern stimuli. This suggests the impaired activation of V5/MT may play a role in impaired motion detection in DLB, but the underlying mechanism behind this requires further study.

### Figure Copying and Other Constructional Tasks

Figure copying relies heavily on both the ventral and dorsal visual streams. The ventral “what” stream is required for perception of the features of the object to be copied, and the dorsal “where” stream is required to determine the spatial relationship between those features and to guide the subject’s movements to create that representation. However, unlike perceptual tasks which rely solely on the encoding and interpretation of a visual image, constructional tasks additionally require executive function to plan the drawing and motor function to draw it. Given that executive and motor impairment are also prominently affected in DLB, these tasks are less precise assessments of visual function. However, visuoconstruction tasks such as figure copying and clock drawing are frequently used as part of office-based cognitive screening exams such as the Mini-Mental Status Exam (MMSE), Montreal Cognitive Assessment (MOCA), and Mini-Cog, providing the practical advantage of easy administration and widespread availability.

Clock drawing in particular has consistently been shown to be impaired in DLB, both when drawing from memory and when copying the image of a clock [6, 69–71], and poorer clock drawing performance has been shown to predict more rapid progression of dementia [72]. Clock drawing impairment is associated with hypometabolism in the precuneus, middle frontal gyrus, and temporoparietal region including the angular gyrus after controlling for MMSE score [73], which reflects the aspects of visuospatial, motor, and executive function required for this task.

The pentagon copy task, which is part of the MMSE, has also been shown to be impaired in DLB [74–77], and errors in the number of angles drawn are common in mild cognitive impairment (MCI) that later evolves into DLB [78, 79]. Pentagon copying impairment in DLB correlates with hypometabolism throughout the occipital cortex as well as posterior temporo-parietal cortex and small regions of frontal cortex, while in AD there is comparable correlation with temporo-parietal and frontal hypometabolism but no correlation with occipital hypometabolism [80].

Other tests of figure copying including the Bender Gestalt test [81, 82] and Rey-Osterrieth complex figure (ROCF) copying [83] have also been shown to be impaired in DLB, and this impairment has correlates with occipital cortex hypometabolism [84, 85]. A recent study applying both FDG and dopamine transporter (DAT) PET to DLB patients found that while both occipital and lateral parietal hypometabolism and caudate DAT signal correlated with ROCF copy performance, path analysis suggested that the cortical hypometabolism alone causally mediated the relationship [85].

### Visual Impairment in prodromal DLB

Studies of visual perception in prodromal DLB or MCI due to presumed Lewy body pathology (MCI-LB) have yielded inconsistent results. Several recent studies found mild abnormalities in subtests of the Visual Object and Space Perception (VOSP) battery in DLB compared to other mild cognitive impairment groups [79, 86, 87], but others have not [88–90]. Errors in figure copying have also been found in patients with isolated REM behavior disorder up to a year prior to DLB diagnosis [91]. Overall, this is an emerging area in need of further research, and these discrepancies may be due to differences in the specific populations and how prodromal DLB-related states were defined.

### Visual hallucinations and illusions

Visual hallucinations (VH) are one of the core clinical features of DLB (Table 1) and a common presenting symptom [92–94], with one key study estimating 72% of DLB patients to have visual hallucinations [95]. Visual hallucinations are much more common than auditory hallucinations, in contrast to the pattern seen in primary psychotic disorders [96]. Patients with DLB can experience complex visual hallucinations of people and animals as well as other visual phenomena, such as visual illusions and brief passage hallucinations [4]. Visual hallucinations have been reported to resolve with eye closure in DLB [97], suggesting that activation of the primary visual cortex is a necessary prerequisite for hallucination formation. The importance of the visual system in visual hallucinations is exemplified by the Charles Bonnet syndrome, a phenomenon in which patients with vision loss due to ophthalmic disease develop visual hallucinations in the absence of neurologic or psychiatric disease. Visual hallucinations in the Charles Bonnet syndrome and DLB have several features in common, although hallucinations due to eye disease are more likely to be simpler phenomena than the complex hallucinations often seen in DLB [98]. Eye disease has been associated with an increased risk of hallucinations in PD [99, 100], suggesting that ophthalmic disease may represents an opportunity to prevent or reduce symptom burden. However, further work is needed to elucidate the relationship between anterior visual pathway impairment and hallucinations in DLB specifically.

Formed visual hallucinations (e.g. seeing faces, animals, or people) in DLB are associated with deficits in visuoperception, abstraction, and attention [52, 101–106]. However, one study found that only verbal memory impairment (and not visual impairment) was predictive of non-hallucinating DLB patients developing new visual hallucinations in the future [104]. Interestingly, while a recent study of patients with DLB and PDD showed complex visual hallucinations correlating with this multidomain cognitive impairment, minor hallucinations (illusions, passage hallucinations, presence hallucinations) were not correlated with cognitive impairment in any domain [102]. They also found that while complex hallucinations were associated with multiple visual and non-visual network disruptions (ventral visual stream, salience network, and default mode network), minor visual hallucinations were associated with decreased connectivity between primary visual areas, ventral visual stream, and brainstem. This raises the hypothesis that disruption in visual networks may be the driver of minor visual hallucinations, but that complex hallucinations require broader disruption involving these other cortical networks.

Perfusion SPECT studies in DLB with and without visual hallucinations have consistently found that visual hallucinations correlate with occipital hypoperfusion, with some studies also showing hypoperfusion in anterior cingulate, orbitofrontal, parietal, and superior temporal cortical regions [107–111]. Metabolic (FDG-PET) and functional (fMRI) connectivity analyses found that patients with DLB who have visual hallucinations show decreased connectivity in both visual and attention systems [32, 112–114]. However, this is not universal, as one functional connectivity analysis showed DLB patients with visual hallucinations had altered connectivity in attention but not visual networks, as well as associated white matter structural abnormalities in association tracts [115]. Structural-electrophysiological connectivity network analysis has found that DLB and PDD patients with hallucinations have both reduced electrophysiological connectivity in the ventral visual network and decreased structural connectivity between visual cortex and subcortical structures (thalamus and nucleus basalis) [28]. Overall, this suggests that dysfunction in visual systems is one driver of visual hallucinations, but other regions and systems, including attention networks and subcortical structures, appear to play key roles as well.

Pareidolia, an illusion wherein an image of one object is misinterpreted as another (for example, mistaking a coat rack for a person), is a symptom that frequently co-occurs with hallucinations in DLB. This can be tested using complex visual scenes (scene pareidolia), or by assessing whether patients perceive faces or objects in a black-and-white noise pattern (noise pareidolia). Of the two, noise pareidolia is more strongly associated with visual hallucinations and neuropsychological impairment in DLB [116, 117]. However, a recent study found that noise pareidolia impairment in DLB did not correlate with occipital cortex hypoperfusion but instead weakly correlated with frontal, parietal, and cingulate cortical hypoperfusion [118]. A recent study using factor analysis to assess relationships between scene pareidolia and other behavioral factors seen in DLB found distinct associations with both 1) visual hallucinations and cognitive fluctuations and 2) visual processing, suggesting both factors may be independently playing a role in pareidolic illusions [119].

Phosphenes can be induced by transcranial magnetic stimulation (TMS), and this has been used as a model to manipulate the brain to produce false visual perceptions analogous to hallucinations. However, there was no difference in phosphene threshold elicited by TMS to the visual cortex in patients with DLB compared to healthy controls, and only a weak relationship between phosphene threshold and hallucination severity in DLB [120, 121]. There was also no significant difference in electrographic cortical response to visual cortex TMS in patients with DLB or PDD, either with or without visual hallucinations [122]. However, patients with DLB who had visual hallucinations showed less activation of the dorsal attention network (DAN) in response to TMS network stimulation, raising the possibility that impairment in this attention network may play a role in visual hallucinations in DLB [122].

Overall, this evidence suggests that visual hallucinations in DLB may result from interplay between visual system dysfunction and other systems, including attention, association, abstract reasoning, and the default mode network.

### Outcomes and quality of life associated with visual dysfunction in DLB

While a considerable amount of research has focused on describing visual dysfunction in DLB and identifying its functional and anatomic correlates, the effects of visual dysfunction on patient-reported outcomes and quality of life have not been well studied. Patients frequently complain of misjudging objects, even in MCI due to presumed Lewy body disease that has not yet progressed to dementia [123–125]. Validated vision-related quality of life instruments such as the VFQ-25 have not been used, though they do not capture visual dysfunction due to neurologic disease as they were developed primarily for patients with reduced visual acuity due to ophthalmic disease (e.g. macular degeneration, cataracts). A 10-item neurological supplement to the VFQ-25 has been developed and validated in other conditions, but this has not been applied to DLB [126].

In the general older adult population, visual impairment is associated with an increased risk of falls through multiple mechanisms, including effects on maintaining posture and navigating stairs. Patients with DLB are already at risk of falls due to parkinsonism and postural instability, and because spatial perception and navigation are also affected, this may further increase the risk of falls. Notably, performance on figure copying tests is associated with fall risk in DLB even when adjusting for other fall risk factors such as visual hallucinations, parkinsonism, and cognitive fluctuations [127].

Driving is another key area where visual dysfunction may have major implications. In a systematic review of the relationship between cognitive assessments and driving in dementia in the general population [128], the Visual Object and Space Perception battery was consistently associated with poor performance in driving simulations across studies [129–131]. A study of simulated driving performance in mild DLB found a strong correlation between driving rating score and performance on the visuospatial but not object subsets of the VOSP [132]. In on-road driving assessments, nearly one in three patients with DLB or PDD failed their driving assessment, primarily due to difficulties with positioning on the road, merging with traffic, and turning left [133, 134].

### Treatment

Evidence-based treatments for visual dysfunction in DLB, especially to improve quality of life and prevent falls and injury, are lacking. The cholinergic neurotransmitter system is prominently affected in DLB, including alterations in cholinergic networks, and acetylcholine transmission is particularly reduced in the occipital lobes [135–141]. However, while cholinesterase inhibitors such as donepezil may reduce visual hallucinations, improve global cognitive function, and even increase occipital lobe perfusion [142], other visual symptoms do not appear responsive to these changes. Specifically, tests of object recognition and visual agnosia did not improve in a randomized controlled trial of donepezil [143]. Trials of galantamine and rivastigmine [144, 145] did not report on primary visuoperceptual measures.

Because of the known relationship between visual function and hallucinations, it has been hypothesized that improving visual acuity through ophthalmologic interventions such as refraction or cataract surgery may reduce hallucinations. Preliminary support for this comes from small case series of patients with non-DLB dementias [146], but more extensive studies are lacking. Physical and occupational therapy interventions to prevent falls commonly include recommendations to maximize contrast and lighting in addition to clearing pathways of obstacles. Further data is needed to clarify how clinicians should best approach driving safety in patients with DLB who have visuoperceptual deficits, especially since standard office-based cognitive assessments do not predict driving performance well. Possibilities to consider for stratifying driving risk include on-road driving assessments, comprehensive neuropsychological assessments, and ride-alongs from support persons to assess driving safety.

### Comparison to Alzheimer disease

DLB is the second most common neurodegenerative cause of dementia after AD. Distinguishing between the two can be challenging, especially in earlier stages of disease when parkinsonism and hallucinations may be mild, but this differential is important especially as amyloid-specific therapies for AD become available. In these cases, testing visuoperception and visuoconstruction can be helpful for clarifying the diagnosis. Compared to AD, DLB is characterized by greater visuospatial impairment and less memory impairment, especially early in disease [5, 6, 71, 147, 148]. In one study, a combination of worse performance on trail-making and the Rey-Osterrieth Complex Figure copy and better performance on the Boston Naming and Auditory Verbal Learning tests best differentiated DLB from AD [83]. A more recent clinical-pathological study found that the Fragmented Letters test was the single best test to distinguish autopsy-confirmed DLB from AD, and that Fragmented Letters test score correlated with the density of Lewy bodies on histopathology [57]. Comparing figure copying and delayed figure recall can also be helpful. AD patients may have difficulty drawing a clock from memory, but this improves when asked to copy a clock from an image they can actively see image. In contrast, DLB patients have difficulty with both copying and spontaneous recall of images [6, 69–71].

## Conclusion

Visual dysfunction is a core feature of DLB, including impairments in contrast and color discrimination, object and form recognition, and spatial orientation and motion. Patients frequently report misjudging objects and distances, and deficits can be illustrated through simple constructional tasks such as figure copying. While treatment options are limited, addressing visuospatial impairment is an important part of a multidisciplinary approach to fall prevention, and improving vision may reduce the burden of hallucinations and other adverse outcomes in DLB, though further research is needed.
